# Retraction: The Increase in IL-1β in the Early Stage of Heatstroke Might Be Caused by Splenic Lymphocyte Pyroptosis Induced by mtROS-Mediated Activation of the NLRP3 Inflammasome

**DOI:** 10.3389/fimmu.2020.01863

**Published:** 2020-07-24

**Authors:** Frontiers Editorial Office

The journal retracts the 11 December 2019 article cited above.

Following the publication of the article, concerns regarding the Figures were addressed to the journal and its Editorial Board. It was confirmed that FACS plots in Figures 4C, 4D, 5, and 9 had been duplicated. Confronted with this concern, the corresponding author confirmed that inappropriate methods had been used to obtain the relevant data and that the images had been manipulated. As a result of this, the scientific conclusions of the manuscript may be unreliable. The article is therefore being retracted.

The authors concur with the retraction and sincerely regret any inconvenience this may have caused to the reviewers, editors, and readers of Frontiers in Immunology.

